# TRPM7 underlies cadmium cytotoxicity in pulmonary cells

**DOI:** 10.1007/s00204-025-04058-4

**Published:** 2025-05-15

**Authors:** Leonor Correia, Alexey Shalygin, Anna Erbacher, Joanna Zaisserer, Thomas Gudermann, Vladimir Chubanov

**Affiliations:** 1 Walther-Straub Institute of Pharmacology and Toxicology, LMU Munich, Munich, Germany; 2 Comprehensive Pneumology Center, German Center for Lung Research (DZL), Munich, Germany

**Keywords:** Cadmium, Magnesium, Zinc, Calcium, NS8593, VER155008

## Abstract

TRPM7 is a kinase-coupled ion channel that exhibits high activity in the immune and epithelial cells of different organs, including the lung. Electrophysiological studies have established that the TRPM7 channel displays high permeability to Mg^2+^, Zn^2+^, and Ca^2+^, as well as trace metal cations. While the critical role of TRPM7 in the cellular balance of Mg^2+^, Zn^2+^, and Ca^2+^ is well-documented, its contribution to the cellular uptake of trace metal cations, frequent respiratory pollutants, remains unclear. Here, we performed an electrophysiological assessment of pulmonary A549 cells revealing endogenous TRPM7 currents, which were eliminated by knockout (KO) of the *TRPM7* gene using the CRISPR/Cas9 approach or by administration of NS8593 and VER155008, two structurally unrelated inhibitors of the TRPM7 channel. Unlike prior studies with various cell lines showing that *TRPM7* KO mutation induces cell growth arrest, we observed that A549 cells maintained normal viability after genetic and pharmacological inactivation of TRPM7. Consequently, we used A549 cells to examine the impact of Cd^2+^ on cell viability and found that *TRPM7* KO mutation and both pharmacological agents mitigated the Cd^2+^ cytotoxicity. Analogous to A549 cells, electrophysiological analysis of mouse primary alveolar type 2 (ATII) cells revealed endogenous TRPM7 currents and Cd^2+^ exposure reduced the cell viability of ATII cells in a TRPM7-dependent fashion. Hence, the TRPM7 channel contributes to Cd^2+^ cytotoxicity in pulmonary cells and can serve as a therapeutic target to alleviate the toxic effects of trace metal exposure.

## Introduction

The transient receptor potential cation channel, subfamily M, member 7 (TRPM7), is a bifunctional protein containing a transmembrane ion channel segment linked to a cytosolic protein kinase domain (Ryazanov et al. 1999; Fleig and Chubanov 2014; Chubanov et al. 2018; Chubanov et al. 2024). Electrophysiological studies revealed that TRPM7 forms a constitutively active channel, which is regulated by several factors, including cytosolic Mg^2+^ and Mg-ATP and membrane lipid phosphatidylinositol-4,5-bisphosphate (PIP_2_) (Nadler et al. 2001; Runnels et al. 2001, 2002; Demeuse et al. 2006; Ferioli et al. 2017; Schmidt et al. 2022). The TRPM7 channel displayed a high permeability to divalent cations, with the following preference for the main divalent cations: Zn^2+^  > Mg^2+^  > Ca^2+^ (Monteilh-Zoller et al. 2003; Schmitz et al. 2003; Li et al. 2006; Li et al. 2007, Mederos et al. 2008).

Several pharmacological compounds were identified as modulators of the TRPM7 channel, which were found to be instrumental in mapping the cellular roles and therapeutic potential of TRPM7 (Fleig and Chubanov 2014; Chubanov et al. 2017; Chubanov et al. 2018; Chubanov and Gudermann 2020; Chubanov et al. 2024). Among other entities, NS8593, waixenicin A, FTY720, VER155008, and CCT128930 represent the most potent inhibitors of the TRPM7 channel (Zierler et al. 2011, Chubanov et al. 2012, Qin et al. 2013, Hofmann et al. 2014, Schafer et al. 2016, Faouzi et al. 2017, Guan et al. 2021, Kollewe et al. 2021, Rossig et al. 2022). Recently, cryogenic electron microscopy (cryo-EM) analysis of TRPM7 revealed the ligand-binding site and molecular mechanisms underlying the inhibitory effects of NS8593, VER155008, and CCT128930 (Nadezhdin et al. 2023, 2024).

TRPM7 is a ubiquitously expressed channel, and its functional role has been extensively investigated in a broad range of cultured cells, animal disease models and genetic association studies in humans (Fleig and Chubanov 2014; Chubanov et al. 2018; Chubanov et al. 2024). One of the most prominent outcomes of these studies is that the loss of TRPM7 function leads to a reduction of cellular contents of Mg^2+^, Zn^2+^, and Ca^2+^, suggesting that the TRPM7 channel mediates the membrane transport of all divalent cations, and, therefore, can be defined as a divalent cation-selective channel (Schmitz et al. 2003, Chubanov, Waldegger et al. 2004, Penner and Fleig 2007, Jin et al. 2008, Ryazanova et al. 2010, Jin et al. 2012, Abiria et al. 2017, Krishnamoorthy et al. 2018, Gupta et al. 2023, Bosman et al. 2024, Egawa et al. 2024). In support of this concept, enterocyte-specific inactivation of *Trpm7* in mice caused severe organismal deprivation of Zn^2+^, Mg^2+^, and Ca^2+^ with the highest impact on Zn^2+^ homeostasis, and this defect was incompatible with early postnatal development of the mutant pups (Mittermeier et al. 2019).

In addition to the main divalent cations, electrophysiological experiments demonstrated that the TRPM7 channel is also highly permeable to trace metal cations, including Co^2+^, Ni^2+^, Ba^2+^, Mn^2+^, Sr^2+^, and Cd^2+^ (Monteilh-Zoller et al. 2003; Li et al. 2006; Li et al. 2007; Tashiro et al. 2014; Kozak and Antosiewicz 2023). However, the physiological relevance of this TRPM7 characteristic has not been investigated in depth. In one study, MG-63 osteoblasts were used to elucidate mechanisms of metal-associated periprosthetic hypoxia (Römmelt et al. 2019). These experiments suggested that TRPM7 can regulate the uptake of Co^2+^ in MG-63 cells, causing HIF-1α upregulation (Römmelt et al. 2019). In another study, A549 cells were utilized to identify candidate transporters responsible for Mn^2+^ uptake in respiratory cells, with TRPM7 being suggested as a Mn^2+^ transporter (Heilig et al. 2006).

The TRPM7 channel has been considered a possible mechanism of cellular uptake of Cd^2+^, a highly toxic and carcinogenic pollutant (Luevano and Damodaran 2014, Genchi et al. 2020), but these studies delivered contradictory results. In osteoblast-like MG-63 and MC3T3-E1 cells, Cd^2+^ exposure led to cell growth arrest, which was mitigated by siRNA *TRPM7* silencing or TRPM7 inhibition, suggesting the involvement of the TRPM7 channel (Lévesque et al. 2008; Martineau et al. 2010). However, in Jurkat T cells, administration of Cd^2+^ suppressed the proliferation of treated cells independently of TRPM7 activity (Mellott et al. 2020). Thus, further studies are necessary to clarify the role of TRPM7 in Cd^2+^ cytotoxicity.

Here, we investigated the contribution of the TRPM7 channel in Cd^2+^ cytotoxicity of pulmonary cells because, in addition to the gastrointestinal tract, the lung is considered the prime target for Cd^2+^ exposure, especially among smokers (Luevano and Damodaran 2014, Genchi et al. 2020, Kozak and Antosiewicz 2023). We found that A549 cells and primary alveolar type 2 (ATII) cells express relatively large endogenous TRPM7 currents and exhibit a high sensitivity to Cd^2+^ administration. Genetic or pharmacological inactivation of the TRPM7 channel attenuated the effect of Cd^2+^ on the viability of A549 cells. TRPM7 inhibitors also mitigated Cd^2+^ cytotoxicity in primary ATII cells. These results support the concept that the TRPM7 channel represents the entry mechanism for toxic metal cations in pulmonary cells.

## Results

### Electrophysiological characterization of the TRPM7 channel in A549 cells

**Fig. 1:**
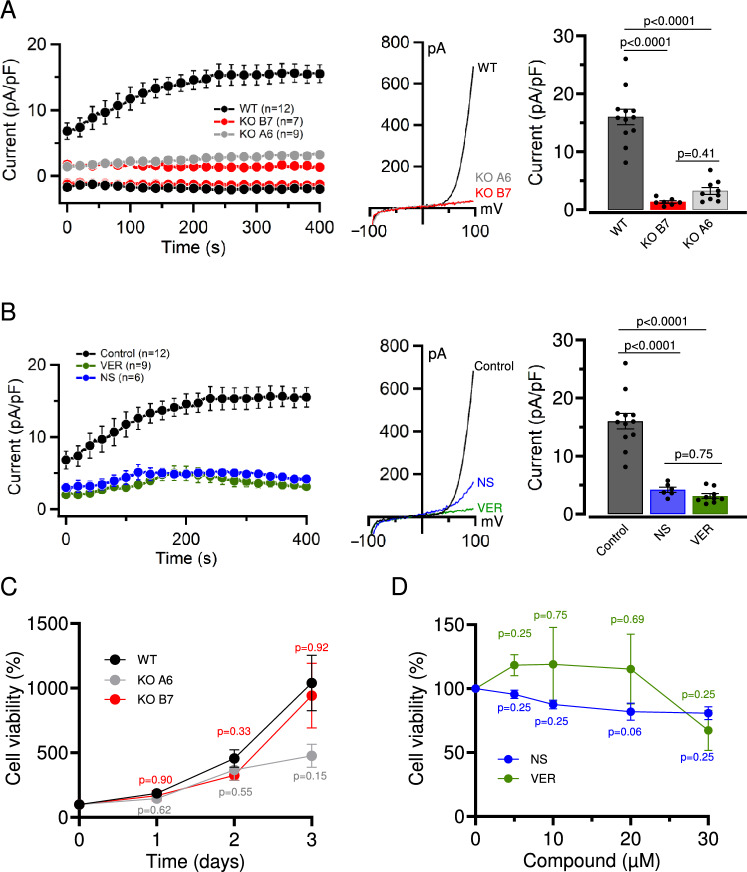
Genetic and pharmacological targeting of TRPM7 in A549 cells. **A** The impact of *TRPM7* KO mutation on TRPM7 currents in A549 cells. *Left panel:* Whole-cell currents were measured at −80 and +80 mV over time in WT (WT) and two *TRPM7* KO (KO A6 and KO B7) A549 cell lines. Data are mean ± SEM; *n*, the number of cells examined. *Middle panel:* Representative current−voltage (I−V) relationships obtained at 400 s in measurements illustrated on the *Left panel*. *Right panel:* Bar graphs of current amplitudes at +80 mV (400 s) illustrated on the *Left panel*. Data are mean ± SEM; *n*, the number of cells examined. The *p* values are shown for ANOVA. **B** The effect of NS8593 or VER155008 on TRPM7 currents in WT A549 cells. Whole-cell currents were measured at −80 and +80 mV over time in the absence (Control) and the presence of 10 μM NS8593 (NS) or 10 μM VER155008 (VER). Data are mean ± SEM; *n*, the number of cells examined. *Middle panel:* Representative current−voltage (I−V) relationships obtained at 400 s in measurements illustrated on the *Left panel*. *Right panel:* Bar graphs of current amplitudes at + 80 mV (400 s) illustrated on the *Left panel*. Data are mean ± SEM; *n*, the number of cells examined. The *p* values are shown for ANOVA. **C** Proliferation rate of WT (WT) and two *TRPM7* KO (KO A6 and KO B7) A549 cell lines. The cells were cultured for 3 days and the initial cell density (day 0) was accounted as 100%. Data are mean ± SEM of *n* = 3 independent experiments. The *p* values are shown for ANOVA comparison of WT versus KO data points. **D** Viability of WT A549 cells maintained for 24 h at different concentrations of NS8593 or VER155008. Cell densities in the absence of pharmacological agents were accounted as 100%. Data are mean ± SEM of *n* = 3 independent experiments. The *p* values are shown for a one-sample *t-*test

Among other in vitro models, the pulmonary A549 cells are frequently used to study the impacts of environmental pollutants and noxious agents, such as cytostatic drugs (Balis et al. 1984; Sakagami 2006; Sporty et al. 2008). Therefore, we asked whether A549 cells are suited to elucidate the role of the TRPM7 channel in the effect of toxic trace cations like Cd^2+^. In patch-clamp measurements, the induction of the TRPM7 channel activity is commonly achieved by removing cytosolic Mg^2+^ using EDTA-containing intracellular solutions (Nadler et al. 2001; Runnels et al. 2001, 2002; Demeuse et al. 2006; Ferioli et al. 2017; Schmidt et al. 2022). We applied this approach to measure endogenous TRPM7 currents in A549 cells (Fig. 1A). The wild-type (WT) A549 cells displayed cation currents, which gradually increased during the first ∼3 min of recordings and remained stable afterwards. The current−voltage (I−V) relationship of fully developed currents was characterized by a steep outward rectification and tiny inward currents at the negative membrane potentials, resembling the features of the TRPM7 channel (Nadler et al. 2001; Runnels et al. 2001, 2002; Demeuse et al. 2006; Ferioli et al. 2017; Schmidt et al. 2022).

To verify the role of TRPM7 in these currents, we performed patch-clamp measurements with A549 cells containing *TRPM7* knockout (*TRPM7* KO) mutations (Fig. 1A). We relied on the CRISPR/Cas9 approach to introduce a deletion in exon 5, resulting in a frame-shift mutation in *TRPM7.* Because clonal selection procedures can affect cell phenotypes, two alternative *TRPM7* KO A549 cell lines were generated (clones A6 and B7). Patch-clamp measurements with *TRPM7* KO A6 and B7 A549 cells revealed that both cell lines were entirely devoid of TRPM7 currents (Fig. 1A).

Because *TRPM7* KO mutations permanently eliminate channel and kinase activities of TRPM7, we asked whether the acute application of NS8593 and VER155008, potent inhibitors of the TRPM7 channel (Chubanov et al. 2012, Rossig et al. 2022; Nadezhdin et al. 2024), can serve as an alternative approach to inactivate the TRPM7 channel in A549 cells. We observed that exposure of WT A549 cells to 10 µM NS8593 or 10 µM VER155008 completely suppressed the TRPM7 channel (Fig. 1B). Hence, the pharmacological agents replicated the impact of *TRPM7* KO mutations on endogenous TRPM7 currents in A549 cells.

In previous studies, *TRPM7* KO mutations in various cells caused a cell growth arrest, which could be restored by a cell culture medium containing additional Mg^2+^ (Schmitz et al. 2003, Ryazanova et al. 2010, Chubanov et al. 2016, Krishnamoorthy et al. 2018, Mittermeier et al. 2019, Chubanov and Gudermann 2020, Schutz et al. 2021, Gupta et al. 2023, Egawa et al. 2024). Unexpectedly, we observed that *TRPM7* KO mutation did not affect the viability of both *TRPM7* gene-deficient A549 cell lines maintained in the regular culture medium (Fig. 1C). Next, we cultured WT A549 cells in the presence of different concentrations of NS8593 or VER155008 for 24 h. We observed that cell growth was modestly reduced by 5−30 µM NS8593 compared to untreated A549 cells, but these changes were not statistically significant (Fig. 1D). Analogously, we did not detect a substantial cytotoxic effect of VER155008 on WT A549 cells. We concluded that genetic or pharmacological inactivation of the TRPM7 channel in A549 cell lines could be achieved without a significant impact on cell viability.

### The role of TRPM7 in Cd^2+^ cytotoxicity in A549 cells

**Fig. 2:**
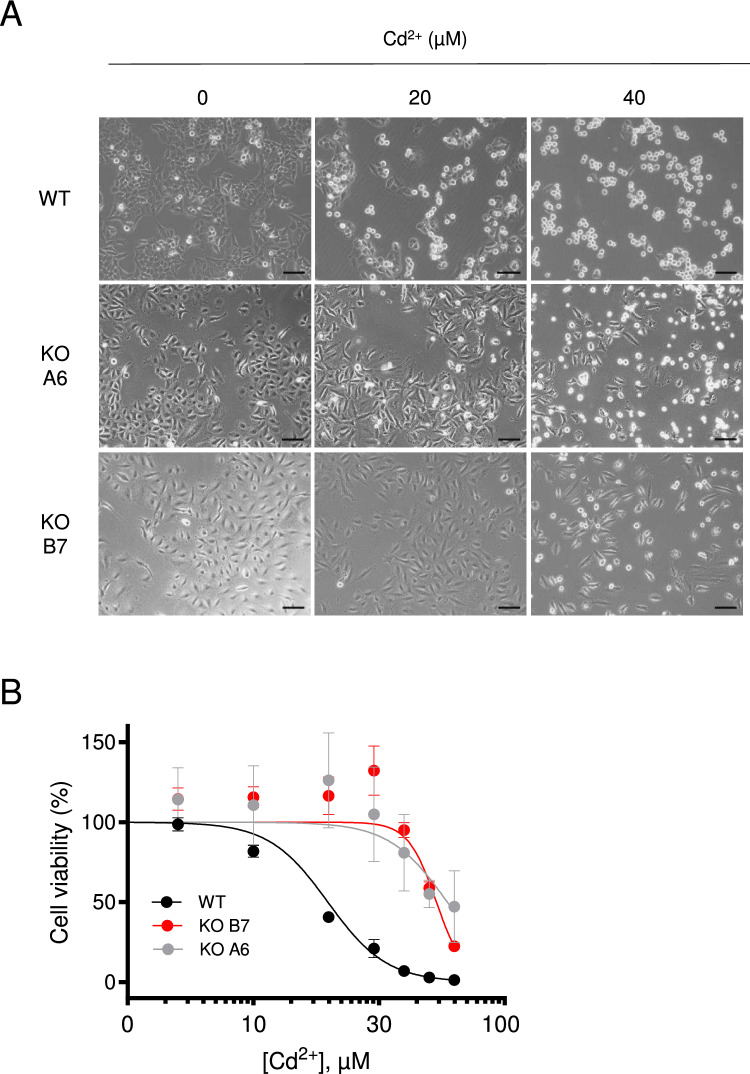
Assessments of Cd^2+^ cytotoxicity in A549 cells. **A** Representative phase-contrast images of WT (WT) and two *TRPM7* KO (KO A6 and KO B7) A549 cell lines maintained for 24 h in the absence or presence of 20 and 40 µM Cd^2+^. Scale bars are 100 µm. **B** Concentration-dependent effects of 24 h Cd^2+^ exposure on the viability of WT (WT) and two *TRPM7* KO (KO A6 and KO B7) A549 cell lines. The density of cells incubated without Cd^2+^ was accounted as 100%. Data are mean ± SEM of *n* = 3 independent experiments. The Hill equation was fitted to determine LC_50_ and the Hill slope (Table 1)

It is well-documented that exposure of cells to Cd^2+^ led to proliferation arrest (Genchi et al. 2020). Given the high Cd^2+^ permeability of the TRPM7 channel (Monteilh-Zoller et al. 2003; Li et al. 2006; Li et al. 2007; Tashiro et al. 2014), we asked whether the TRPM7 channel plays a role in such cytotoxicity effect in A549 cells. To this end, we cultured WT and *TRPM7* KO A549 cells in the absence or presence of different concentrations of Cd^2+^ in the cell culture medium (Fig. 2). We found that 24 h exposure of WT A549 cells to 20 µM Cd^2+^ reduced cell density and resulted in accumulation of cellular debris, cell rounding, and loss of adherence indicative of cell death (Fig. 2A). These changes were more pronounced if the cells were treated with 40 µM Cd^2+^ (Fig. 2A). However, both *TRPM7* KO A549 cell lines showed a remarkably reduced sensitivity to Cd^2+^ treatments (Fig. 2A).

Consequently, we quantified the toxic effects of 24 h Cd^2+^ treatment on the proliferation rate of A549 cells using the Neutral Red assay (Fig. 2B). We found a concentration-dependent reduction of cell viability of WT A549 cells with an LC_50_ value of 19.81 µM (Table 1). In accordance with cell imaging results, two alternative *TRPM7* KO A549 cell lines displayed a remarkably reduced cell viability upon the Cd^2+^ exposure (Fig. 2B). The LC_50_ values for *TRPM7* KO A6 and B7 cells were, respectively, 59.18 µM and 53.56 µM (Table 1), suggesting that the TRPM7 channel contributes to cellular responses to Cd^2+^ when this cation is present in the low µM range (Fig. 2B).

**Table 1:** Cd^2+^ cytotoxicity in WT and *TRPM7* KO A549 cell lines

Genotype	LC_50_ (µM)	Hill slope
WT	19.81 ± 0.50	– 3.71 ± 0.38
*TRPM7* KO A6	59.18 ± 15.23 (*p* = 0.0003)*	– 3.74 ± 0.90 (*p* = 0.3021)*
*TRPM7* KO B7	53.56 ± 0.79 (*p* < 0.0001)*	– 7.69 ± 0.21 (*p* = 0.4146)*

^*^p values obtained from *F*-test for *n* = 3 independent experiments shown in Fig. 2B

**Fig. 3:**
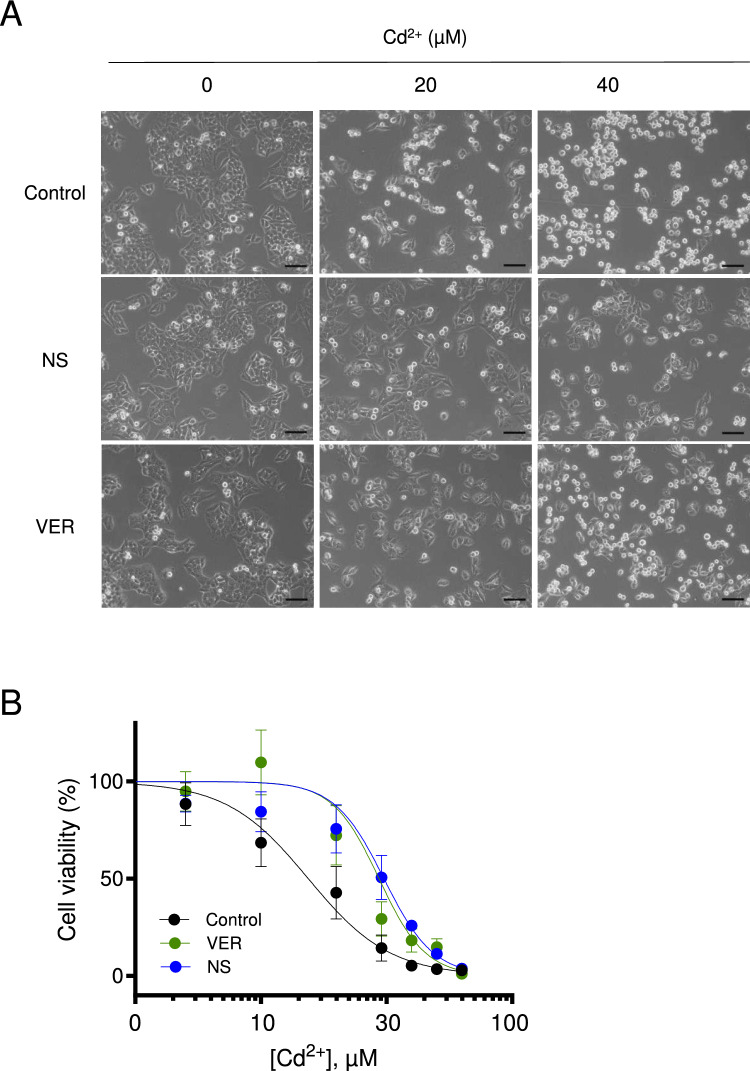
Effects of TRPM7 inhibitor on Cd^2+^ cytotoxicity in A549 cells. **A** Representative phase-contrast images of DMSO-treated (Control) WT A549 cells or cells exposed for 24 h to 20 µM NS8593 (NS) and 20 µM VER155008 (VER) in the absence or presence of 20 and 40 µM Cd^2+^. Scale bars are 100 µm. **B** Concentration-dependent effects of 24 h Cd^2+^ exposure on the viability of DMSO-treated (Control) WT A549 cells or the cells exposed to 20 µM NS8593 (NS) and 20 µM VER155008 (VER). The density of the cells incubated without Cd^2+^ was accounted as 100%. Data are mean ± SEM of *n* = 3 independent experiments. The Hill equation was fitted to determine LC_50_ and the Hill slope (Table 2)

Next, we studied whether acute inactivation of TRPM7 by NS8593 and VER155008 can also ameliorate the toxic effect of Cd^2+^ on WT A549 cells (Fig. 3). We observed that the 24 h treatment by 20 and 40 µM Cd^2+^ elicited characteristic morphological changes in control A549 cells (WT cells treated by DMSO), but this effect was not observed in the cells cultured in the presence of 20 µM NS8593 (Fig. 3A). Administration of 20 µM VER155008 also reversed morphological changes in the cells exposed to 20 µM Cd^2+^ and, to a lesser degree, in the cells treated with 40 µM Cd^2+^ (Fig. 3A).

Consequently, we determined the dose-dependent effects of Cd^2+^ on the viability of WT A549 cells in the absence or presence of TRPM7 inhibitors (Fig. 3B). The calculated LC_50_ was 15.83 µM for control (DMSO-treated) A549 cells, whereas co-application of 20 µM NS8593 or 20 µM VER155008 resulted in LC_50_ values of 32.70 µM and 32.59 µM, respectively (Table 2). These results further support the notion that the TRPM7 channel plays a role in the toxic effects of Cd^2+^ in A549 cells.

**Table 2:** Cd^2+^ cytotoxicity in WT A549 cells treated by TRPM7 inhibitors

Treatment	LC_50_ (µM)	Hill slope
Control (DMSO)	15.83 ± 4.14	– 2.70 ± 1.33
NS8593	32.70 ± 1.04 (*p* < 0.0001)*	– 4.67 ± 0.98 (*p* = 0.0055)*
VER155008	32.59 ± 10.02 (*p* = 0.0271)*	– 5.56 ± 0.65 (*p* = 0.3035)*

^*^p values obtained from *F*-test for *n* = 3 independent experiments shown in Fig. 3B

### TRPM7 contributes to Cd^2+^ cytotoxicity in primary alveolar type 2 (ATII) cells

**Fig. 4:**
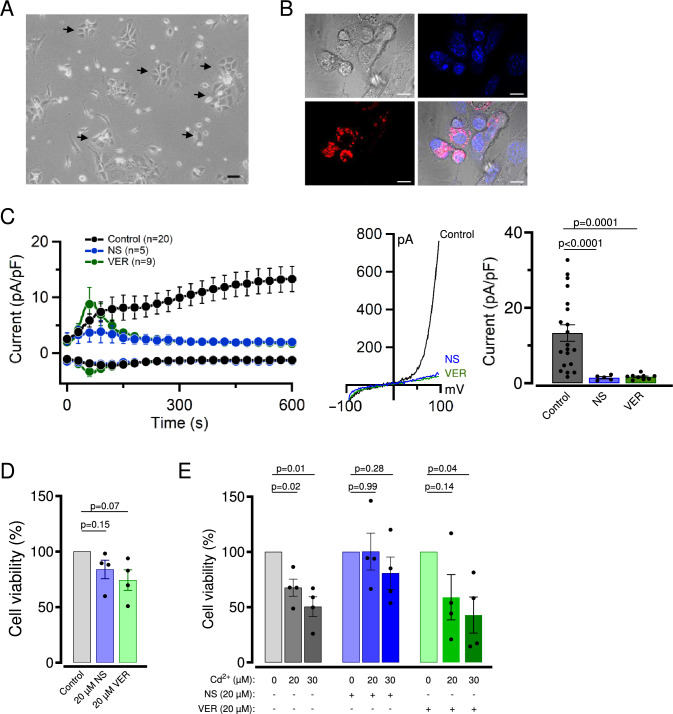
Effects of NS8593 and VER155008 on primary ATII cells. **A** Representative phase-contrast images of ATII cells isolated from mouse lungs and maintained for 2 days in ATII cell culture medium. Arrows indicate characteristic colonies formed by primary ATII cells. The scale bar is 100 µm. **B** Representative DIC and confocal images of living ATII cells labelled by LysoTracker Red (red) and Hoechst 33342 (blue) and their overlay are shown. Scale bars are 10 μm. *n* = 2 independent isolations. **C** The effect of NS8593 (NS) and VER155008 (VER) on TRPM7 currents in ATII cells. *Left panel:* Whole-cell currents were measured at −80 and +80 mV over time in the absence (Control) and the presence of 5 μM NS8593 (NS) or 5 μM VER155008 (VER). Data are mean ± SEM; *n*, the number of cells examined. *Middle panel:* Representative current−voltage (I−V) relationships obtained at 600 s in measurements illustrated on the *Left panel*. *Right panel:* Bar graphs of current amplitudes at +80 mV (600 s) illustrated on the *Left panel*. Data are mean ± SEM; *n*, the number of cells examined. The *p* values are shown for ANOVA. **D** Viability of ATII cells maintained for 24 h in the presence of 20 μM NS8593 (20 μM NS) or 20 μM VER155008 (20 μM VER) or equal volume of DMSO (Control). Cell densities in the absence of pharmacological agents were accounted as 100%. Data are mean ± SEM of *n* = 4 independent experiments. The *p* values are shown for a one-sample *t*-test. **E** Effects of TRPM7 inhibitor on Cd^2+^ cytotoxicity in A549 cells. Experiments were conducted and analyzed similar to (**D**) in the absence and presence of 20 μM or 30 μM Cd^2+^. Data are mean ± SEM of *n* = 4 independent experiments. The *p* values are shown for a one-sample *t-*test

Given the notable contribution of the TRPM7 channel to Cd^2+^ cytotoxicity in A549 cells, we asked whether TRPM7 is implicated in the toxic effects of Cd^2+^ in more physiological settings. Because A549 cells are frequently used as an in vitro correlate of pulmonary alveolar type 2 (ATII) cells (Balis et al. 1984; Sakagami 2006; Sporty et al. 2008), we conducted electrophysiological experiments to detect endogenous TRPM7 currents in primary ATII cells. The ATII cells were isolated from the lungs of adult mice and cultured for 3−4 days, followed by the assessment of TRPM7 currents using the patch-clamp approach. In line with previous studies (Gereke et al. 2012, Weber et al. 2020), the isolated ATII cells displayed an epithelial-like morphology and formed dense colonies with a characteristic shape after 2−3 days of cell culture (Fig. 4A). The primary ATII cells were further verified using the fluorescent dye LysoTracker Red, a well-established marker of lamellar bodies in ATII cells (Haller et al. 1998; Van der Velden et al. 2013). As expected, the LysoTracker Red signal was accumulated in numerous vesicular compartments of ATII cells (Fig. 4B).

The electrophysiological analysis of ATII cells revealed membrane currents resembling endogenous TRPM7 currents found in A549 cells (Fig. 4C). Analogous to the results with A549 cells, TRPM7 currents in ATII cells were fully diminished by the exposure of ATII cells to either NS8593 or VER155008 (Fig. 4C). However, we noted that the VER155008-treated ATII cells exhibited a transient increase of small inward and outward currents of unknown origin (Fig. 4C). We concluded that the TRPM7 channel is functionally expressed in primary ATII cells.

We next investigated whether the application of NS8593 and VER155008 interferes with the cell growth of primary ATII cells. We found that the administration of 20 µM NS8593 did not affect the growth of ATII cells (Fig. 4D). Application of 20 µM VER155008 resulted in a modest reduction in the proliferation of ATII cells, but this effect was found to be statistically insignificant (Fig. 4D). Consequently, we examined both TRPM7 inhibitors in their ability to protect ATII cells from the cytotoxicity effects of 20 and 30 µM Cd^2+^ (Fig. 4E). We found that both doses of Cd^2+^ significantly reduced the viability of control (DMSO-treated) ATII cells (Fig. 4E). Co-administration of NS8593 normalized the cell growth of the Cd^2+^-treated ATII cells (Fig. 4E). However, the protection effect of VER155008 was only registered for 20 µM Cd^2+^ (Fig. 4E).

Collectively, our findings suggest that the TRPM7 channel critically contributes to the cytotoxic action of Cd^2+^ on primary ATII cells.

## Discussion

The lung represents one of the prime organs in organismal Cd^2+^ poisoning, especially among smokers (Jumarie 2002; Tai et al. 2022), prompting us to investigate whether the cytotoxic effects of Cd^2+^ in respiratory cells are linked to the TRPM7 channel activity. We demonstrated that the TRPM7 channel is functionally expressed in A549 cells and primary type 2 pneumocytes isolated from mouse lungs. We also showed that loss of the TRPM7 channel activity results in the significantly reduced effect of Cd^2+^ on the viability of these cells, indicating that the TRPM7 channel underpins Cd^2+^ cytotoxicity in pulmonary cells.

Cd^2+^ is a frequent pollutant in soil, water, and air, eliciting toxic and carcinogenic effects upon organismal poisoning [45]. With respect to the respiratory system, exposure to Cd^2+^ is a risk factor for chronic obstructive pulmonary disease (COPD), tissue fibrosis, and lung cancers (Kwon et al. 2006; Oh et al. 2014; Nawrot et al. 2015; Cetintepe et al. 2019; Cui et al. 2021; Lee et al. 2022; Wang et al. 2023; Peng et al. 2024; Prasad et al. 2024). However, little is known about the mechanisms underlying Cd^2+^ uptake in respiratory cells.

In electrophysiological experiments, the TRPM7 channel was found to be highly permeable for trace metal cations, and consequently, it has been proposed as a possible mechanism of cellular uptake of toxic exogenous cations, including Cd^2+^ (Monteilh-Zoller et al. 2003). Previously, two in vitro models were used to examine the role of TRPM7 in Cd^2+^ cytotoxicity. Initially, MG-63 and MC3T3-E1 osteoblasts were investigated because Cd^2+^ disrupts bone metabolism and induces osteoporosis (Lévesque et al. 2008; Martineau et al. 2010). Serum-starved MG-63 cells were found to be highly sensitive to Cd^2+^ added to the culture medium with the LC_50_ of 18 µM. Adding extracellular Mg^2+^ or TRPM7 inhibitor 2-APB mitigated the Cd^2+^-induced growth arrest and inhibited ^109^Cd^2+^ uptake. In similar experimental conditions, Cd^2+^ exposure of serum-starved MC3T3-E1 cells also induced a cytotoxic effect with the LC_50_ of 9 µM, which was significantly ameliorated by Mg^2+^ supplementation. Moreover, ^109^Cd^2+^ uptake in MC3T3-E1 cells was significantly reduced upon application of channel inhibitors 2-APB and Gd^3+^ or siRNA *TRPM7* silencing (Lévesque et al. 2008; Martineau et al. 2010).

Another group investigated the role of TRPM7 in the cytotoxicity effects of trace metal cations in Jurkat T cells using cell viability as a readout (Mellott et al. 2020). Interestingly, 0.4 mM Cd^2+^ nearly completely suppressed the proliferation rate of Jurkat T cells, and this effect was not affected by varying external levels of Mg^2+^ or by the administration of TRPM7 inhibitors, like NS8593 and FTY720 (Mellott et al. 2020). Unfortunately, the LC_50_ values of Cd^2+^ were not determined in this study. Nevertheless, the author interpreted these results as TRPM7 not contributing to Cd^2+^ cytotoxicity in Jurkat T cells (Mellott et al. 2020). The discrepancy between this finding and results obtained with MG-63 and MC3T3-E1 cells can be attributed to differences in cell culture conditions, Cd^2+^ doses examined, and the cell-specific role of TRPM7 in Cd^2+^ uptake. Hence, further studies are required to sort out these issues.

In the present work, we used patch-clamp techniques to show that A549 cells and primary ATII cells exhibit characteristic endogenous TRPM7 currents. In A549 cells with *TRPM7* KO mutations, such currents were not detectable. Moreover, TRPM7 currents were entirely suppressed in A549 cells and primary ATII cells after applying NS8593 and VER155008, two structurally unrelated inhibitors of the TRPM7 channel. Primary ATII cells also tolerated the treatment by NS8593 and VER155008 well. Of note, despite previous reports linking the loss of TRPM7 function to Mg^2+^-dependent cell growth (Schmitz et al. 2003, Chubanov, Waldegger et al. 2004, Penner and Fleig 2007, Jin et al. 2008, Ryazanova et al. 2010, Jin et al. 2012, Abiria et al. 2017, Krishnamoorthy et al. 2018, Gupta et al. 2023, Bosman et al. 2024, Egawa et al. 2024), we did not observe significant changes in the proliferation of A549 cells after inactivation of *TRPM7* in A549 cells. Hence, A549 cells can be very instrumental in mapping the role of TRPM7 in the effects of cytotoxic agents, such as environmental pollutants.

When exposed to Cd^2+^, WT A549 cells revealed dose-dependent suppression of cell growth with the LC_50_ of ~ 20 µM, while two clones carrying *TRPM7* KO mutations exhibited ~ threefold increased LC_50_ values. In line with these findings, the acute inactivation of the TRPM7 channel using NS8593 or VER155008 also significantly mitigated the effect of Cd^2+^ on WT A549 cells and primary ATII cells. These results suggest that the TRPM7 channel is a critical mediator of Cd^2+^ toxicity in pulmonary cells and highlight TRPM7 as a target to prevent tissue damage linked to Cd^2+^ poisoning.

Apart from TRPM7, other TRP channels have also been suggested to contribute to cellular responses to Cd^2+^ exposure. Thus, TRPA1 was shown to mediate Cd^2+^ influx in mouse sensory neurons, and this process is linked to acute pain induction by Cd^2+^ administration to mice (Miura et al. 2013). TRPV5 and TRPV6, two highly Ca^2+^-selective channels, were also found to be permeable to Cd^2+^ (Kovacs et al. 2011; Kovacs et al. 2013). Moreover, transient overexpression of TRPV5 or TRPV6 increased the antiproliferative effect of Cd^2+^ on HEK293 cells (Kovacs et al. 2011; Kovacs et al. 2013). Hence, the results of the present study reinforced the idea that TRP channels represent the crucial route of Cd^2+^ entry in mammalian cells.

## Materials and methods

### A549 cells

A549 cells were maintained at 37 °C and 5% CO_2_ in Ham’s F-12 K medium supplemented with 10% FBS (both from Thermo Fisher Scientific, Waltham, MA, USA), 100 µg/ml streptomycin and 100 U/ml penicillin (Merck, Darmstadt, Germany).

A CRISPR/Cas9 approach was used to generate A549 cells carrying frame-shift mutations in the *TRPM7* gene (Ubigene, Guangzhou, China). The exon 5 of *TRPM7* was targeted by transfection of parental wild-type (WT) A549 cells with CRISPR-U vectors encoding the following gRNA nucleotides: CCACGAATCAAGCAGTTGCTTGG and TTGCAGAATGACTTCAGGTTTGG. Genotypes of the obtained clones were examined by PCR-based analysis of genomic DNA extracted with the GenElute mammalian genomic DNA miniprep kit (Merck, Darmstadt, Germany). For PCR reaction, we used Taq Plus Master Mix (Vazyme, Nanjing, China) and two primers 5’-GGAGTCCGCCCCGTGAGG-3’ and 5’-TGACTTCCGCCCCATACTTTCCAACAG-3’ (Eurofins Genomics, Ebersberg, Germany) with the PCR settings: 95 °C 30'', 63 °C 15'', 72 °C 90''. The PCR products were confirmed by sequencing (Eurofins Genomics, Ebersberg, Germany).

Two alternative *TRPM7* KO A549 cell lines have been isolated, herein referred to as *TRPM7* KO B7 and A6. The *TRPM7* KO B7 A549 cells contained an 80-bp deletion (CATGGGGGCATGCAGAAATTTGAGCTTCACCCACGAATCAAGCAGTTGCTTGGAAAAGGTCTTATTAAAGCTGCAGTTAC) in both *TRPM7* alleles. The *TRPM7* KO A6 cells exhibited the same 80-bp deletion in one allele and a 79-bp deletion (TGGGGGCATGCAGAAATTTGAGCTTCACCCACGAATCAAGCAGTTGCTTGGAAAAGGTCTTATTAAAGCTGCAGTTACA) in the second allele of *TRPM7*. In silico analysis revealed that both deletions led to frame-shift mutations.

### Primary mouse alveolar type 2 (ATII) cells

ATII cells were isolated from the lungs of adult *C57BL/6 J* mice, as reported earlier (Weber et al. 2020). Handling of animals and all experimental procedures were performed in accordance with the guidelines of the European Union for the use of animals. The mice were killed by cervical dislocation, and the lungs were flushed via a catheter through the right ventricle with 0.9% NaCl solution (Carl Roth, Karlsruhe, Germany). Tissue dissociation procedures were performed using an isolation medium based on Dulbecco’s Modified Eagle Medium—Low Glucose (Merck, Darmstadt, Germany) with 2% GlutaMAX (Thermo Fisher Scientific, Waltham, MA, USA), 10 mM HEPES (PanReac AppliChem, Darmstadt, Germany), 100 µg/ml streptomycin and 100 U/ml penicillin (Merck, Darmstadt, Germany). Lungs were inflated through the trachea with 1.5 ml of dispase in HBSS (50 U/ml) (Corning, Corning, NY, USA) followed by 0.3 ml of the isolation medium containing 1% low-melting-point agarose (Carl Roth, Karlsruhe, Germany) and incubated for 1 h at room temperature in dispase solution. Subsequently, lung lobes were dissociated in the isolation medium with 0.04 mg/ml DNase I (PanReac AppliChem, Darmstadt, Germany), filtered through 100 μm, 20 μm, and 10 μm nylon filters (Sefar, Heiden, Switzerland), and centrifuged for 10 min at 200×*g*. Cell pellets were resuspended in the isolation medium, plated on CD45- and CD16/32-antibody coated (BD Biosciences, Franklin Lakes, NJ, USA) culture dishes, and incubated at 37 °C for 30 min for a negative selection of immunocytes and lymphocytes. Non-adherent cells were collected, seeded on uncoated dishes, and left to incubate at 37 °C for 50 min for negative selection of fibroblasts. Non-adherent ATII cells were collected and cultured for 3–4 days in the isolation medium supplemented with 10% FBS (Thermo Fisher Scientific, Waltham, MA, USA) at 37 °C and 5% CO_2_.

### Confocal laser-scanning microscopy

ATII cells were cultured for 3 days on glass-bottom cell culture dishes (World Precision Instruments, Sarasota, FL, USA). Cells were washed with ATII cell culture medium and incubated with 100 nM LysoTracker Red and 1 µg/ml Hoechst 33342 (both from Thermo Fisher Scientific, Waltham, MA, USA) in culture medium for 30 min at 37 °C and 5% CO_2_. The culture medium was removed and freshly applied two times. After 30 min incubation, differential interference contrast (DIC) and confocal images of LysoTracker Red and Hoechst 33342 fluorescence were obtained with the confocal laser-scanning microscope LSM 880 AxioObserver (Carl Zeiss, Oberkochen, Germany). We used a C-Apochromat 63x/1.2 W objective, 561 nm or 405 nm excitation wavelengths and 566–690 nm or 410–587 nm band-pass filters for LysoTracker Red and Hoechst 33342 fluorescence, respectively. The acquired images were analysed using the ZEN 3.0 SR software (Carl Zeiss, Oberkochen, Germany).

### Electrophysiological techniques

Patch-clamp experiments were performed as reported previously with a few modifications (Ferioli et al. 2017; Rossig et al. 2022; Schmidt et al. 2022; Nadezhdin et al. 2023). Whole-cell currents were measured using an EPC10 patch-clamp amplifier and PatchMaster software (Harvard Bioscience, Holliston, MA, USA). Voltages were corrected for a liquid junction potential of 10 mV. Currents were elicited by a ramp protocol from –100 mV to +100 mV over 50 ms acquired at 0.5 Hz and a holding potential of 0 mV. Inward and outward current amplitudes were extracted at –80 mV and +80 mV and were normalized to the cell size as pA/pF. Capacitance was measured using the automated capacitance cancellation function of EPC10. Unless stated otherwise, the extracellular solution contained (in mM) 140 NaCl, 2.8 KCl, 3 CaCl_2_, 10 HEPES-NaOH, and 11 glucose (all from Merck, Darmstadt, Germany). Solutions were adjusted to pH 7.2 using an FE20 pH meter (Mettler Toledo, Columbus, OH, USA) and 290 mOsm using a Vapro 5520 osmometer (Wescor Inc, South Logan, UT, USA). Patch pipettes were made of borosilicate glass (Science Products, Hofheim, Germany) and had a resistance of 2–3.7 MΩ when filled with the standard Mg^2+^-free intracellular pipette solution containing (in mM) 120 Cs-glutamate, 8 NaCl, 10 Cs-EGTA, 5 Cs-EDTA, and 10 HEPES-CsOH (all from Merck, Darmstadt, Germany). The intracellular solution was also adjusted to pH 7.2 and 290 mOsm. The results are presented as the mean ± standard errors of the means (SEM). Data showed a normal distribution. For multiple comparisons, ANOVA followed by Dunnett's multiple comparison test was used (GraphPad Prism software 10.4.1). Significance was accepted at *p* ≤ 0.05.

### Assessment of cell viability

To study the growth rate of WT and *TRPM7* KO A549 cells (Fig. 1C, D), the cells of each genotype were seeded in a 96-well plate (5–10 × 10^3^ cells/well) in the standard A549 culture medium. After 24 h, the cell culture medium was replaced by medium with or without Cd^2+^ (CdCl_2_; Merck, Darmstadt, Germany) and the cell densities were determined at different time intervals using the Neutral Red assay kit (Abcam, Cambridge, UK) according to the manufacturer’s manual. The cell density in the absence of Cd^2+^ was accounted as 100%. To study the impacts of TRPM7 inhibitors, NS8593 (Tocris, Bristol, UK), VER155008 (Tocris, Bristol, UK) or equivalent volumes of DMSO were added to the cell culture medium with or without additional Cd^2+^. The density of the DMSO-treated cells in the absence of Cd^2+^ was accounted as 100%. For a statistical comparison, ANOVA followed by Dunnett's multiple comparison test or a one-sample *t*-test (GraphPad Prism software 10.4.1) were used as indicated in the figure legend. Significance was accepted at *p* ≤ 0.05.

Dose-dependent Cd^2+^ cytotoxicity in A549 cells (Figs. 2B, 3B; Tables 1, 2) was analyzed using the following equation (GraphPad Prism 10.4.1):

$$CD(c)={CD}_{min}+\frac{{CD}_{max}-{CD}_{min}}{1+{10}^{(logLC50-c)*h}}$$

with *CD* being the normalized cell density at a given concentration *c* of Cd^2+^; *CD*_*min*_, the minimal cell density; *CD*_*max*_, the highest cell density; *LC*_*50*_, the half-lethal concentration; *h*, the Hill slope. Fitting of dose–response curves was performed using GraphPad Prism 10.4.1. A statistical comparison of *LC*_*50*_ and *h* was performed by the extra sum-of-squares *F*-test (GraphPad Prism 10.4.1). Significance was accepted at *p* ≤ 0.05.

To study Cd^2+^ effects on the survival of ATII cells (Fig. 4D, E), the cells were isolated from 3 mice, pooled, and seeded in a 96-well plate in the ATII culture medium, treated with Cd^2+^ in the absence or presence of TRPM7 inhibitors and examined similarly to A549 cells. A statistical comparison was performed using a one-sample *t-*test (GraphPad Prism 10.4.1). Significance was accepted at *p* ≤ 0.05.

## Data Availability

Derived data supporting the findings of this study are available from the corresponding authors upon request.
